# Nurturing resilience in American Indian/Alaska Native preschool children: the role of cultural socialization, executive function, and neighborhood risk

**DOI:** 10.3389/fpsyg.2023.1279336

**Published:** 2023-11-30

**Authors:** Alexis Merculief, Shannon Lipscomb, Megan M. McClelland, G. John Geldhof, Monica Tsethlikai

**Affiliations:** ^1^Human Development and Family Studies, Oregon State University, Corvallis, OR, United States; ^2^Sanford School of Social and Family Dynamics, Arizona State University, Tempe, AZ, United States

**Keywords:** cultural socialization, language socialization, resilience, executive function, preschool, neighborhood risk, Indigenous, AI/AN

## Abstract

**Introduction:**

American Indian and Alaska Native (AI/AN) children possess numerous cultural assets, yet higher exposures to neighborhood risks (e.g., lack of housing, crime) may present barriers to healthy cognitive development, including executive function (EF). Cultural socialization may promote resilience and support children’s early cognition, but this has not been adequately studied. The present study examined the effects of neighborhood risk and cultural socialization on EF for AI/AN preschool children.

**Method:**

Parents/caregivers of 768 AI/AN preschoolers from the 2015 AI/AN Head Start Family and Community Experiences (FACES) Study rated neighborhood risk via two scales: “Neighborhood Problems” and “Environmental Conditions,” and cultural socialization practices via two scales: cultural activities and tribal language activities. Children’s EF was measured directly using the Pencil Tap Task and the Leiter-R attention subscale.

**Results:**

Families perceived neighborhood risks as relatively low, and overall risk did not predict children’s EF. However, higher average language socialization was significantly related to higher EF, as were two specific language activities (encouraging children to learn their tribal language, making sure children heard their tribal language) and two cultural activities (playing AI/AN games, participating in tribal ceremonies), controlling for neighborhood risk.

**Discussion:**

Findings suggest some aspects of cultural socialization may promote resilience among AI/AN preschoolers by supporting early EF. Mechanisms may include increased spiritual, social, and cultural connections, and practice with EF skills during cultural games. Future research should partner with AI/AN communities to investigate culturally grounded EF interventions and reevaluate measures of neighborhood risk to promote resilience and connectedness for AI/AN children.

## Introduction

American Indian and Alaska Native (AI/AN) children are immersed in communities with rich cultural traditions and strong relational connections. Yet, they also carry the generational effects of historical trauma and poverty, resulting in barriers to healthy development including greater risks in the neighborhood or community environment (e.g., crime, poor housing quality, lack of access to healthy food; Bauer et al., 2012; Burnette and Figley, 2016; Austin et al., 2020; Baldwin et al., 2020). These risks present persistent, daily stressors for families (Schaefer-McDaniel, 2009), and accumulation of these risks predicts detrimental health outcomes (Schaefer-McDaniel, 2009; Carroll-Scott et al., 2013; Muñoz et al., 2020), although less is known about influences on early cognition. Since AI/AN youth as young as 13 years are at higher risk than the general U.S. population for substance use, depression, suicide, and dropping out of school (Hawkins et al., 2004; Mmari et al., 2009; Myhra and Wieling, 2014; Baldwin et al., 2020; Morrell et al., 2020), early prevention and intervention efforts are needed. Such efforts include boosting individual resilience factors like children’s executive function (EF) skills, as well as resilience processes already embedded in AI/AN children’s communities like culture and language. The present study examined relations between neighborhood risk and EF for AI/AN preschool children and investigated cultural socialization as a resilience-promoting factor for the development of EF.

Resilience is sometimes defined as the capacity to adapt to challenges that “threaten the function, survival, or future development of a system” (Masten and Barnes, 2018). Systems involve individual, family, community, sociological, and ecological levels (Ungar, 2021). Furthermore, resilience factors may be promotive (related to healthy development for children in all contexts, including adversity), protective (attenuating negative impacts of adversity on healthy development), or both (Masten, 2018). Emerging Indigenous theories of resilience hold community at the core, with both the source and goal of resilience being community thriving rather than individual adaptation (Hart, 2010). The Indigenous Connectedness Framework and related models (Ullrich, 2019; Ivanich et al., 2023) therefore conceptualize aspects of resilience as “connecting forces” and aspects of risk as “disconnecting forces.” AI/AN children access connecting forces by way of familial, intergenerational, communal, spiritual, and environmental connectedness mechanisms. This manuscript uses an integrative approach (see Garcia Coll et al., 1996), to weave together Indigenous and Western theory and methodology as they inform investigation into cultural socialization as a resilience promoter for EF in the context of risk for AI/AN children. Examples of integration include centering Indigenous theories and emphasizing cultural and historical contexts, while also utilizing Western theories and measures. Thus, we describe resilience resources as flowing between communities and individuals but nevertheless appreciate the practical value of assessing developmental outcomes at the individual rather than communal level.

EF (sometimes referred to as self-regulation) represents a set of foundational cognitive skills, understood to include working memory, inhibitory control, and cognitive flexibility. These skills allow children to regulate their behavior and emotions in alignment with their social contexts (McClelland et al., 2015). In AI/AN communities, EF and related skills are sometimes conceptualized as moving beyond emotional and behavioral regulation to achieve balance in all aspects of being- mind, body, spirit, and emotions (Tsethlikai et al., 2018). Early childhood represents a developmental window of rapid growth in EF, a window which also represents a period of enhanced vulnerability to environmental contexts, including both risk and resilience factors (Best and Miller, 2010). Strong EF in early childhood is itself an individual resilience factor, in that it predicts future academic achievement (Ursache et al., 2012; McClelland et al., 2013), positive peer relationships (Farley et al., 2014), lower risk of depression (Papadakis et al., 2006; Joormann et al., 2016), and fewer risk-taking behaviors (Romer et al., 2009; Pharo et al., 2011). EF also functions as a protective factor for cognitive development in the face of risks like low socioeconomic status (SES). For example, Sektnan et al. (2010) found that in a national sample, children from underserved backgrounds (e.g., low SES and underserved races/ethnicities) scored lower on literacy and math in first grade; yet children from similar backgrounds with higher EF skills in preschool scored about half a standard deviation higher on these measures of early academics. However, less is known about how EF development is also shaped by other protective and promotive factors for resilience, particularly in the context of risks embedded in the neighborhoods of young AI/AN children.

The neighborhood environment can have profound impacts on healthy development, depending on which supportive or adverse influences are present (Garcia Coll et al., 1996). Examples of adverse neighborhoods (referred to as “neighborhood risks” in this manuscript) include lack of housing or green spaces, low proximity to health services, and residential crowding (Sallis et al., 2011; Carroll-Scott et al., 2013; Rollings et al., 2017), as well as social aspects like crime, substance abuse, and low social support (Schaefer-McDaniel, 2009; Sallis et al., 2011; French et al., 2014). Less is known about neighborhood risks in AI/AN communities, and it should be noted that the term “neighborhood” may not reflect the nuances of tribal reservations. Reservations represent not only geographical areas but sovereign nations, often culturally homogenous communities with a shared history. Nevertheless, the term “neighborhood” is used in the present study to maintain consistency with measures in the data and existing literature. There is some literature to suggest that AI/AN families, like others from underserved racial and ethnic groups, face unequal access to high-quality neighborhoods due to systemic injustices such as housing discrimination (Osypuk et al., 2009; Sharkey and Elwert, 2011) and forced relocation from traditional lands (Mmari et al., 2009; Burnette and Figley, 2016). AI/AN communities are also at greater risk for living in areas with high crime (Burnette and Figley, 2016; Baldwin et al., 2020), low access to healthy foods (Bauer et al., 2012), and greater exposure to drugs and other substances (Morrell et al., 2020).

Neighborhood risks are associated with chronic stress, poor mental health (Schaefer-McDaniel, 2009; Muñoz et al., 2020), poor physical health (Carroll-Scott et al., 2013; Muñoz et al., 2020), substance abuse (Morrell et al., 2020), and low academic achievement (Schaefer-McDaniel, 2009; Sastry and Pebley, 2010; Sharkey and Elwert, 2011). This adversity may also impact cognition due to chronic stress on prefrontal cortex functioning (Sandi, 2013), especially when the stress exceeds coping resources (Lazarus and Folkman, 1984). However, less is known about specific influences of neighborhood risk on EF in early childhood, especially in AI/AN communities. To our knowledge, only two studies report associations between neighborhood risks (e.g., safety, aesthetics, and social cohesion) and lower childhood EF or related skills (Caughy and O’Campo, 2006; Roy et al., 2014). The study by Caughy and O’Campo (2006) showed that neighborhood poverty predicted lower simultaneous processing skills for African American preschool children, and the study by Roy et al. (2014) documented a negative impact of moving to high-poverty neighborhoods on self-regulation for children three to 6 years old. However, neither study focused on AI/AN children, and neither examined the influence of cultural resilience factors on EF in the context of risk.

Culture may be especially important for EF in early childhood, as EF (or self-regulation) skills are developed through an inherently social process. Family and community provide safe relationships and model co-regulation for children (Carlson, 2009). Core aspects of culture and language may further support co-regulation, build strong identity, and connect children to healthy relationships; but little is known about direct relations between AI/AN culture and EF. Since “AI/AN” does not represent one race but over five-hundred tribes, a singular definition of “AI/AN culture” does not exist. Still, researchers have identified several common values: ethnic or tribal pride, sense of belonging, connection to all living things, balance and harmony, communal responsibility, shared history of overcoming trauma, and traditional activities (such as dance, songs, art, and storytelling; Stevens et al., 1999; House et al., 2006; Powers, 2006). Tribal language is frequently included as a vital component of culture, as it preserves culture and connectedness among community members (Whitbeck et al., 2004; House et al., 2006). Lasting effects of historical trauma, assimilation, and the boarding school era (where AI/AN children were forcibly removed from their families) has led to what many call the “hibernation” of tribal languages (Campbell and Evans-Campbell, 2011; De Costa, 2021). However, while present-day parents may no longer speak their tribal language, many tribes have directed resources to reawakening these sleeping languages by teaching them in schools as well as offering classes for families to learn tribal languages together.

Cultural connection and identity may promote and protect resilience via better physical and mental health (Bhui et al., 2005; Mmari et al., 2009; Wexler, 2009; Burnette and Figley, 2016), even in the face of adversity. Strong AI/AN cultural identity is sometimes associated with better mental health and higher self-esteem (Markstrom et al., 2011; Williams et al., 2018; Baldwin et al., 2020), as well as lower substance abuse and less risky behavior for adolescents and adults (Herman-Stahl et al., 2003; Baldwin et al., 2020). For example, AI/AN ethnic pride was associated with adherence to antidrug norms with AI/AN adolescents in one study (Kulis et al., 2001), and another study demonstrated cultural connectedness was associated with increased reasons for life (or life purpose, a protective factor for suicide) for Alaska Native adolescents (Mohatt et al., 2011). Conversely, other studies find that AI/AN identity may be associated with higher rates of substance use (Markstrom et al., 2011), lower academic achievement (Powers, 2006), and more risky behavior (Hawkins et al., 2004; Silmere et al., 2006) among adolescents. The inconsistent measurement of culture could be influencing these conflicting results (House et al., 2006; Markstrom et al., 2011), yet it is also possible stronger AI/AN affiliation increases exposure to racism and historical trauma. The conflicting results may also be related to low SES, which is often associated with lower EF and related skills (Sektnan et al., 2010; Rea-Sandin et al., 2021). While many prior studies do control for family SES, it may be that community-wide poverty and other systemic risks impact EF in ways that mask the positive influence of cultural resources, but this association remains unclear.

Even less is known about these resilience processes in early childhood, which represents a period of cultural socialization, wherein children observe and learn from their caregiver’s cultural identity as they become socialized in the customs and traditions of their community (Phinney and Ong, 2007). While community is critical for this process, the family is especially central to, and a strong predictor of, a child’s eventual cultural identity (Phinney and Ong, 2007; Markstrom et al., 2011). Consequently, parent/caregiver cultural socialization practices have the power to serve as connecting forces and imbue AI/AN children with rich features of resilience such as: a sense of purpose, motivation, connection their ancestors, hope, optimism, and connections to healthy adults who can help them co-regulate their behaviors and emotions (McCaslin, 2009; Williams et al., 2018; Guo et al., 2021). Moreover, cultural socialization coincides with the developmental window of rapid growth in EF in preschool. However, while research has explored cultural *differences* in the manifestation of EF skills (e.g., Rea-Sandin et al., 2021), there has been little investigation into the relation between cultural identity/socialization and EF, especially in early childhood. The closest related literature focuses on AI/AN tribal identity in pre-adolescent and adolescent children, with some studies suggesting a protective influence of cultural identity on academic achievement (Whitbeck et al., 2004), school involvement, and educational persistence (Powers, 2006). Even less is known about how cultural socialization may serve as a promotive or protective resilience factor for healthy cognitive development in the face of adversity (e.g., neighborhood risks).

Thus, the present study examined whether parent/caregiver cultural socialization practices served as resilience factors (i.e., connectedness mechanisms) by promoting emerging EF skills for AI/AN children in the context of neighborhood risk. The first research question investigated the relation between neighborhood risk (e.g., parent/caregiver perceptions of safety, presence of alcohol/drugs, sense of social support) and EF, hypothesizing that higher risk would predict lower EF (Caughy and O’Campo, 2006; Roy et al., 2014; Ursache et al., 2022). The second research question examined whether cultural socialization (e.g., participation in cultural activities, exposure to tribal language) promoted EF in the presence of neighborhood risk. To fill a significant gap in the field and increase theoretical knowledge around cultural socialization, this construct was measured both cumulatively (sum cultural activities, average language activities) and individually by examining relations between specific cultural socialization activities (e.g., “participating in tribal ceremonies”) and EF. While these item-level analyses were exploratory in nature, it was hypothesized that higher overall cultural socialization (e.g., more participation in community activities, higher average frequency of tribal language use) would be related to higher EF, controlling for neighborhood risk.

## Method

### Study design

Data come from the 2015 AI/AN Head Start Family and Community Experiences (FACES) study, a periodic evaluation of the characteristics, family experiences, and development of children attending reservation-based Head Start programs in the United States. To address the multitribal national context, all measures used in 2015 AI/AN FACES (Bernstein et al., 2018; Malone et al., 2018) were evaluated and/or adapted for use with AI/AN families by a diverse workgroup that included researchers, community partners, Head Start partners, and government employees- many of whom were Indigenous, and others with experience working with Indigenous communities (Sarche et al., 2022). The study recruited children from 21 randomly selected Region XI Head Start programs across the United States during fall and spring of the 2015–2016 school year, and created sampling weights to represent all AI/AN children enrolled in Region XI that same year. Data for this study were collected in Fall of 2015 (family demographics, cultural socialization) and spring of 2016 (direct child assessments of EF, neighborhood risk). Fall and Spring data points are considered to collectively represent the preschool period; this study is cross-sectional rather than longitudinal.

### Participants

Participants included 768 children identifying as AI/AN, alone or in combination with another race/ethnicity, and their parents or caregivers (To protect the confidentiality of participating individuals and tribal communities, AI/AN FACES did not document tribal affiliation). Approximately 50% were female, and average age in fall of preschool was 4.17 years (*SD* = 0.56). Children exclusively attended tribal Head Start programs, where over half (65.9%) received at least some instruction in their tribal language and one-third (29.05%) used culturally based curriculum. At home, a large majority (94.1%) of families spoke English as their primary language, with 42.4% also speaking their tribal language at home. Nearly one quarter (27.7%) of children lived in a multigenerational household with a grandparent or great-grandparent, as is common with AI/AN families (Red Horse, 1997). Nearly 80% of respondents to the family survey were mothers, an additional 8.5% were fathers, 8.4% were grandparents/great-grandparents, and the rest identified with another relation to the child. Families reported an average annual household income between $20,000 and $30,000, with 42.5% of families meeting federal poverty level criteria. Parent/caregiver survey response rates fluctuated between 80 and 100%. Missingness did not depend upon any observed demographic variables.

### Measures

#### Neighborhood risk

Caregivers rated risk in their neighborhood or community via two scales created for AI/AN FACES (for measure development, reliability, and validity see: Bernstein et al., 2018; Malone et al., 2018), both containing items that align with measures of neighborhood risk in prior studies with the general U.S. population. The nine-item “Neighborhood Problems” scale (Table 1) asked participants to rate various neighborhood concerns using a three-point Likert scale where 1 = “not a problem,” 2 = “somewhat of a problem,” and 3 = “big problem.” Example items include: “not enough good housing,” “crime,” and “alcohol and/or drug abuse.” The four-item “Environmental Conditions” scale (Table 1) used a five-point Likert scale from 1 (strongly disagree) to 5 (strongly agree), with higher scores representing more risk, and asked caregivers to assess neighborhood conditions such as: “People around here are willing to help their neighbors,” (reverse coded) and “The place where I live is too noisy or too polluted.” Due to conceptual overlap, for hypothesis testing, the two scales were combined to represent one index: “Neighborhood Risk,” by averaging z-scores for all 13 items (*α* = 0.88, *M* = 0.001, *SD* = 0.66).

**Table 1:** Neighborhood risk index and contributing scales.

	Percent of sample reporting each response option (*n* = 768)			
Neighborhood problems	Not a problem	Somewhat of a problem	Big problem	Missing
Rundown houses/abandoned cars	43.1	30.7	10.3	15.9
Crime	34.0	34.2	15.6	16.2
Police not available	50.0	20.2	13.5	16.3
Public drunkenness/high/stoned	39.1	23.6	21.0	16.4
Broken homes/family breakups	32.9	32.4	18.1	16.5
Physical violence/abuse/neglect	40.5	27.1	15.8	16.7
Alcohol and/or drug abuse	28.5	22.8	32.6	16.2
Not enough good housing	36.1	25.4	22.0	16.5
Not enough jobs	19.7	27.3	36.5	16.5

	Percent of sample reporting each response option (*n* = 768)					
Environmental conditions	Strongly disagree	Disagree	Neutral	Agree	Strongly agree	Missing
Too noisy/polluted	23.8	41.4	10.4	5.7	2.3	16.3
Roads are difficult to drive	16.9	41.7	13.2	9.6	2.9	15.8
Too far from shopping/gas/etc.	13.0	35.7	13.0	16.8	5.6	15.9
People are willing to help neighbors (reverse)	3.8	10.6	25.3	32.4	12.2	15.8
		*N*	*M*	*SD*	Min	Max
**Neighborhood risk index (standardized)**		643	0.001	0.66	−1.12	1.70

#### Executive function (EF)

The present study used both a direct assessment (the Pencil Tap Task; Golden et al., 1982; Diamond and Taylor, 1996) and an assessor rating of child attention (the Leiter-R; Roid and Miller, 1997; Roid et al., 2009), a multi-method strategy that provides complimentary information on early EF (Smith-Donald et al., 2007; McClelland and Cameron, 2012). The Pencil Tap and Leiter-R were correlated at *r* = 0.25 (*p* < 0.001) and analyzed as separate outcomes in the current study due to methodological differences as well as different facets of EF measured (e.g., predominately attention with the Leiter-R versus predominately inhibitory control with the Pencil Tap). Both measures are part of the Preschool Self-Regulation Assessment (PSRA), which demonstrates internal and external validity in preschool samples, including dual-language learners (Smith-Donald et al., 2007; Malone et al., 2018), but to our knowledge have not been utilized with AI/AN children. However, evaluation by a third party found no performance concerns with either of these measures and noted their psychometric strength with the present sample (Malone et al., 2018).

##### Pencil Tap

During the Pencil Tap Task, the assessor taps their pencil (once or twice), and the child must inhibit their natural desire to mimic the assessor and instead do the opposite of what the assessor does. Scores are presented as the percentage of correct responses out of total responses, with higher scores indicating higher EF. Children younger than four years were not assessed with the Pencil Tap (27.9% of the sample), in alignment with developer guidelines (Malone et al., 2018; see Analytic Strategy). Internal reliability for the present sample was *α* = 0.92. Children tapped correctly on average 48.04% of the time (*SD* = 33.05, Range = 0–100).

##### Leiter-R assessor-rated attention

This 10-item scale measures assessors’ subjective ratings of child attention during direct assessments (e.g., Pencil Tap and other measures of early literacy, vocabulary, and mathematics). Behaviors (e.g., “child focuses on task,” and “pays attention during instructions and demonstrations,”) were rated on a three-point Likert scale from 1 (rarely/never) to 3 (usually/always), with higher scores indicating greater attention. Scale reliability was not able to be calculated due to copyright restrictions prohibiting item-level data from the developer, but reports from 2015 AI/AN FACES state reliability as *α* = 0.97 (Bernstein et al., 2018). The average assessor rating of attention on the Leiter-R was 26.12, *SD* = 5.97 (Range = 0–30).

#### Cultural socialization

Cultural socialization was measured via parent/caregiver report using two scales created for AI/AN FACES (for measure development, reliability, and validity see: Bernstein et al., 2018; Malone et al., 2018) that measured children’s participation in six cultural activities and six tribal language activities with their parents/caregivers in the past month. Cultural and language socialization activities were analyzed cumulatively (i.e., sum cultural activities, average language activities) for hypothesis testing. At the same time, each item (activity) represents a distinct cultural experience that reflects more than a single “cultural socialization” factor. To fill a significant gap in the literature on AI/AN cultural socialization, and at the request of members of the AI/AN community, additional models assessed each cultural and language activity independently to focus on specific resilience processes in this initial study of EF development with a preschool-aged AI/AN sample.

##### Cultural socialization activities

The **“**Community Activities with Child” scale asked parents/caregivers whether their children had engaged in six AI/AN cultural activities in their communities in the past month (see Table 2, *α* = 0.71). Examples include: “listened to elders tell stories” and “participated in traditional ceremonies.” On average, parents/caregivers reported their child engaging in 2.30 (*SD* = 1.83) out of six possible cultural activities.

**Table 2:** Cultural and language socialization activities with child (past month).

		Percent of sample (*n* = 768)		
Cultural activities			Yes	No
Listened to elders tell stories			48.96	50.39
Participated in traditional ways			48.70	51.30
Danced/sang/drummed/other activity			44.53	55.47
Worked on traditional arts/crafts			31.64	68.36
Participated in traditional ceremonies			33.98	66.02
Played AI/AN games			21.74	77.21
	*M*	*SD*	Min	Max
**Cultural activities (sum)**	2.30	1.18	0.0	6.0

	Percent of sample (*n* = 768)				
Language activities	Never	Rarely	Sometimes	Often	Always
Spoke tribal language to child	27.0	20.4	25.5	16.7	10.4
Made sure child heard tribal language	17.2	16.7	21.4	25.8	18.9
Encouraged child to learn tribal language	19.7	9.6	16.2	29.4	24.9
Used tribal language in prayers/songs with child	36.7	18.4	20.6	12.8	11.5
Used tribal language in everyday life with child	30.5	20.4	19.1	16.8	13.2
Spoke tribal language with other adults around child	39.8	16.2	19.3	13.5	11.1
		*M*	*SD*	Min	Max
**Language activities (average)**		2.75	1.16	1.0	5.0

##### Language socialization activities

The “Parent’s Tribal Language Use with Child” scale asked parents/caregivers the extent to which they had engaged in six specific language activities with their child in the past month (see Table 2, *α* = 0.91), using a Likert scale ranging from 1 (never) to 5 (very often). Examples include: “spoke tribal language with child,” and “used tribal language in prayers and songs with child.” Average parent/caregiver language socialization was 2.75 (*SD* = 1.16).

#### Covariates

Demographics such as age, sex, and SES are correlated with EF in childhood (Sektnan et al., 2010; Schmitt et al., 2015) and were included as covariates in all models. Annual household income was chosen to represent SES in the present study, with income categories as follows: $0–5,000, $5,001-10,000, $10,001-15,000, $15,001-20,000, $20,001-25,000, $25,001-30,000, $30,001-35,000, $35,001-40,000, $40,001-50,000, $50,001-75,000, and $75,001+. Aspects of shared classroom environment (use of cultural curriculum and tribal language; coded 0 = did not use cultural/language curriculum, 1 = utilized cultural/language curriculum) were also included as covariates to retain focus on family cultural socialization practices rather than socialization provided by Head Start programs. Furthermore, tribal home language (binary; coded as 0 = no tribal language spoken at home, 1 = tribal language spoken at home either as first or second language) was included in models measuring language socialization to focus on parent/caregiver socialization practices above and beyond their own language abilities. Tribal home language was highly correlated with language socialization (*α* = 0.69, see Table 3), but variance inflation factor (VIF) scores were under 2.5, which current literature suggests is not a concern for multicollinearity (James et al., 2017). Out of caution, and at the request of Indigenous research partners and community members, additional analyses assessed the influence of language socialization on EF across tribal home language groups (families who spoke their tribal language at home versus those who did not), and results are reported after main hypothesis testing.

**Table 3:** Correlations between EF, cultural socialization, neighborhood risk, and covariates.

	1	2	3	4	5	6	7	8	9	10	11
1. N. risk index	1.00										
2. Pencil tap	−0.05	1.00									
3. Leiter-R	0.00	0.30***	1.00								
4. Cultural soc.	0.13***	−0.01	−0.03	1.00							
5. Lang. soc.	0.29***	−0.05	−0.02	0.51***	1.00						
6. Class lang.	0.17***	0.00	−0.01	0.06	0.26***	1.00					
7. Class curric.	0.08*	0.04	0.03	0.11**	0.16***	0.17***	1.00				
8. Home lang.	0.30***	−0.05	−0.03	0.33***	0.69***	0.22***	0.18***	1.00			
9. Income	−0.20***	0.05	0.02	−0.05	−0.24***	−0.18***	−0.10***	−0.24***	1.00		
10. Gender	0.03	−0.02	−0.07*	−0.07*	0.02	0.03	0.03	0.02	0.02	1.00	
11. Age	0.12**	0.27***	0.10**	0.10**	0.12***	0.06**	0.09**	0.11**	−0.11**	0.06	1.00

Spearman correlations reported. *p < 0.05; **p < 0.01; ***p < 0.001. N. risk index, neighborhood risk index; Soc., socialization; Lang., language; Curric., curriculum.

## Analytic strategy

### Missing data

Missingness on variables relevant to the present study ranged from 0.3% (Language Activities) to 27.9% (Pencil Tap Task). High missingness on the Pencil Tap was due to children under 4 years of age not being assessed as per the developer’s age guidelines (Analytic Strategy for Models Using the Pencil Tap below). Missingness on this and other relevant constructs (EF tasks, Leiter-R, neighborhood risk, cultural and language socialization, and covariates) did not depend upon any other observed variables. Because some variables had greater than 10% missingness (Bennett, 2009; Dong and Peng, 2013), missing data were handled using full information maximum likelihood (FIML) in all SEM analyses with the Pencil Tap, and maximum likelihood (ML) in hurdle models with the Leiter R, due to software limitations not allowing for FIML with hurdle models.

### Accounting for survey design and shared environments

Analyses for the present study were run using STATA 15 (StataCorp, 2017). A “survey set” command was utilized in which the software stores information regarding complex survey design; including the primary sampling units, strata, and sampling weights (provided by the 2015 AI/AN FACES dataset and recommended for inclusion by the user manual). This method also accounted for shared variance in each level of the sampling design (e.g., Head Start center) to provide accurate standard errors. Intra-class correlations (ICCs) suggested that between-center variation accounted for less than 5% of the variability in EF, but accounted for upwards of 25% of neighborhood risk and 18% of cultural socialization variables. Shared environment regarding classroom characteristics such as use of cultural curriculum and tribal language instruction was also controlled for (see Covariates).

### Analytic strategy for models using the Pencil Tap

Regression analyses were run in an SEM framework to examine neighborhood risk and overall cultural and language socialization as predictors of the Pencil Tap. Subsequent models assessed each cultural activity and language activity independently. All models controlled for family income, age, gender, classroom cultural curriculum use, classroom tribal language instruction, and tribal home language use. As children under 4 years were not assessed using the Pencil Tap in alignment with developer guidelines, sensitivity analyses were run that restricted the sample to children 4 years of age and older. The pattern of results did not change, so data from all participants was retained in the final chosen models.

### Analytic strategy for models using the Leiter-R

Because nearly 50% of the children scored at ceiling (30), the distribution for the Leiter-R Assessor Rated Attention was moderately skewed (−1.73) and kurtotic (5.56). High scores on the Leiter-R assessor ratings are common in preschool populations (Raver et al., 2011; Mathematica Policy Research, 2012; Faldowski et al., 2013), yet the Leiter-R shows construct and predictive validity with other measures of EF and with children’s school readiness outcomes (Smith-Donald et al., 2007; Daneri et al., 2018). For the present study, analytic options to address non-normality were limited due to copyright prohibitions that restricted item-level data. Thus, we adopted an exploratory approach using hurdle models to test hypotheses with Leiter-R sum scores. Hurdle models contain two steps: the first step runs a linear or exponential regression with children who scored 0–29. The second step runs a logistic regression to test independent variables as predictors of group membership: children who scored at ceiling (30) versus those who did not. Because the conditional mean of the Leiter-R had an exponential form, scores were reversed such that 30 = 0, where 0 represented the “lower limit” in the hurdle models. Coefficients reported with hurdle models were reversed back to their original directionality for interpretation. For comparison, ZIP and censored regression models were also considered as an alternative to hurdle models (results available from the first author upon request). These strategies yielded a similar pattern of results, but hurdle models better fit the data and were more parsimonious. All models controlled for family income, age, gender, classroom cultural curriculum use, classroom tribal language instruction, and tribal home language.

## Results

### Neighborhood risk and EF

Families reported perceiving the average risk on the Neighborhood Problems scale as just below “somewhat of a problem.” (1.84, *SD* = 0.62, where 1 = not a problem, 2 = somewhat of a problem, 3 = big problem). The average rating of risk across the four Environmental Conditions items was likewise relatively low: 2.37 (*SD* = 0.69; where 1 = strongly disagree, 5 = strongly agree and higher represents more risk). Contrary to expectations, the Neighborhood Risk Index that combined these two scales was not significantly related to EF via the Pencil Tap (Table 4) or the Leiter-R (Table 5). Of note, while family SES moderately correlated with neighborhood risk (*r* = −0.20, *p* < 0.001; Table 3), SES was not related to either EF measure. However, higher scores on the Neighborhood Risk Index significantly and positively related to higher cultural socialization with both cultural activities (*r* = 0.10, *p* = 0.01) and language activities (*r* = 0.25, *p* < 0.001).

**Table 4:** Neighborhood risk and cultural socialization as predictors of the Pencil Tap.

Predictors	Estimate	SE	95% CI		*p*
			LL	UL	
Neighborhood risk index	−2.19	2.95	−8.49	4.10	0.47
Cultural activities (sum)	0.01	0.70	−1.48	1.50	0.99
Language activities (average)	1.80	1.20	−0.76	4.36	0.15
Age	1.57	0.22	1.10	2.04	0.00
Gender	−2.38	3.09	−8.97	4.20	0.45
Income	1.69	0.63	0.34	3.03	0.02
Class tribal language use	2.46	4.69	−7.53	12.46	0.61
Class cultural curriculum	3.20	2.86	−2.90	9.30	0.28
Tribal home language	−1.57	3.90	−9.89	6.74	0.69

*Analyses account for sampling weights, clustering, and strata due to complex survey design.

**Table 5:** Neighborhood risk and cultural socialization as predictors of the Leiter-R: hurdle models.

Model	Estimate	SE	95% CI		*p*
			LL	UL	
Exponential regression results (0–29)					
Neighborhood risk index	0.10	0.15	−0.22	0.42	0.51
Cultural activities (Sum)	0.00	0.04	−0.09	0.10	0.92
Language activities (Average)	0.10	0.09	−0.09	0.28	0.28
Age	0.01	0.01	−0.01	0.03	0.43
Gender	−0.15	0.09	−0.35	0.05	0.12
Income	0.01	0.02	−0.04	0.05	0.77
Class tribal language use	−0.26	0.21	−0.71	0.19	0.24
Class cultural curriculum	0.11	0.15	−0.20	0.42	0.47
Tribal home language	−0.48	0.19	−0.88	−0.07	0.03
Logistic regression predicting hurdle (30)					
Neighborhood Risk Index	0.12	0.10	−0.09	0.33	0.23
Cultural Activities (Sum)	−0.04	0.03	−0.10	0.02	0.18
**Language activities (Average)**	**0.21**	**0.09**	**0.01**	**0.40**	**0.04**
Age	0.02	0.01	0.00	0.03	0.05
Gender	−0.30	0.14	−0.60	−0.00	0.05
Income	0.03	0.04	−0.04	0.11	0.36
Class tribal language use	−0.34	0.18	−0.73	0.05	0.08
Class cultural curriculum	0.25	0.21	−0.18	0.69	0.24
Tribal home language	−0.32	0.18	−0.70	0.07	0.10

All analyses account for sampling weights, clustering, and strata due to complex survey design. Leiter-R was reverse coded for analyses, coefficient signs have been flipped back to original directionality for interpretation.

### Cultural socialization and EF

#### Cultural activities

The cumulative (sum) measure of cultural activities was not significantly related to EF via the Pencil Tap (Table 4) or the Leiter-R (Table 5). Similarly, four specific cultural activities (including listening to elders tell stories, participating in traditional ways, participating in traditional arts and crafts, and dancing/signing/drumming) also demonstrated nonsignificant associations with EF (see Tables 6, 7). However, two cultural activities did demonstrate significant relations with EF: playing AI/AN games and participating in tribal ceremonies. Playing AI/AN games significantly predicted higher scores on the Pencil Tap Task [*ß* = 10.28, SE (*ß*) = 3.05, *p* = 0.004; Table 6], such that children who played AI/AN games in the past month had 51.53% correct taps [SE (*ß*) = 2.66] compared with 41.26% correct taps [SE (*ß*) = 1.45] for those who did not. Likewise, children who participated in tribal ceremonies with their parent/caregiver were more likely to pass the hurdle (receive a score of 30) on the Leiter R for assessor-rated attention. Children who participated in ceremonies had 29.7% greater odds [95% CI (1.05, 1.61)] of belonging to the highest scoring group than children who did not (Table 7). Participating in ceremonies did not predict the Leiter-R for children who scored 0–29.

**Table 6:** Specific cultural and language activities as predictors of the Pencil Tap task.

Activity			95% CI		*p*
	Estimate	SE	LL	UL	
Listened to elders tell Stories	1.00	2.52	−4.44	6.28	0.72
Participated in traditional ways	0.26	2.44	−4.94	5.47	0.92
Danced/sang/drummed/other activity	−3.16	2.11	−7.65	1.33	0.15
Worked on traditional arts/crafts	1.09	2.50	−4.23	6.41	0.67
Participated in traditional ceremonies	−2.12	4.59	−11.91	7.67	0.65
**Played AI/AN games**	**10.23**	**3.05**	**3.78**	**16.77**	**0.00**
Spoke tribal lang. to child	1.50	1.49	−1.67	4.68	0.33
Made sure child heard tribal lang.	0.20	1.59	−3.12	3.58	0.90
**Encouraged child to learn tribal lang.**	**3.39**	**0.12**	**3.13**	**3.65**	**0.00**
Used tribal lang. in prayers/songs with child	1.26	0.93	−0.72	3.24	0.20
Used tribal lang. in everyday life with child	−0.69	0.79	−2.37	0.99	0.39
Used tribal lang. with other adults around child	−0.37	1.13	−2.44	2.36	0.97

Separate regression analyses were conducted for each cultural or language activity. All models account for sampling weights, clustering, and strata due to complex survey design, and controlled for age, gender, income, classroom cultural curriculum, classroom tribal language use, tribal home language, and neighborhood risk.

**Table 7:** Specific cultural activities as predictors of the Leiter-R assessor-rated attention: hurdle models.

Model	Estimate	SE	95% CI		*p*
			LL	UL	
Exponential regression results (0–29)					
Listened to elders tell stories	0.02	0.13	−0.33	0.07	0.19
Participated in traditional ways	0.01	0.07	−0.15	0.16	0.92
Danced/sang/drummed/other activity	0.12	0.15	−0.19	0.44	0.42
Worked on traditional arts/crafts	0.21	0.15	−0.11	0.52	0.19
Participated in traditional ceremonies	0.17	0.17	−0.19	0.54	0.34
Played AI/AN games	−0.10	0.14	−0.39	0.19	0.48
Logistic regression predicting hurdle (30)					
Listened to elders tell Stories	0.06	0.14	−0.24	0.36	0.67
Participated in traditional Ways	−0.10	0.12	−0.36	0.16	0.43
Danced/sang/drummed/other activity	0.12	0.17	−0.47	0.24	0.50
Worked on traditional arts/crafts	0.02	0.10	−0.20	0.23	0.88
**Participated in traditional ceremonies**	**0.26**	**0.11**	**0.03**	**0.50**	**0.03**
Played AI/AN games	−0.00	0.10	−0.22	0.22	0.99

Separate regression analyses were ran for each cultural or language activity. All models account for sampling weights, clustering, and strata due to complex survey design, and controlled for age, gender, income, classroom cultural curriculum, classroom tribal language use, tribal home language, and neighborhood risk.

#### Language activities

As expected, average participation in tribal language activities was associated with higher EF when measured via the Leiter-R: children whose parents/caregivers engaged in more language socialization activities with them were more likely to pass the “hurdle” on the Leiter-R (Table 5). Average participation in tribal language activities was not significantly related to the Leiter-R for children who scored 0–29, nor did it predict EF via the Pencil Tap. Two specific language activities also significantly related to higher EF: encouraging children to learn their tribal language, and making sure children heard their tribal language. Children whose parents/caregivers encouraged them to learn their tribal language scored higher on the Pencil Tap Task [*ß* = 3.15, SE (*ß*) = 0.95, *p* = 0.01; Table 6], with children whose caregivers stated they “very often” encouraged their child to learn tribal language scoring 49.48% correct taps [SE (*ß*) = 2.32] compared with 36.89% correct taps [SE (*ß*) = 2.37] with for those whose parents/caregivers “never” did. Hurdle models further demonstrated that children whose parents/caregivers made sure they heard their tribal language received higher attention ratings on the Leiter-R [*ß* = 0.14, SE (*ß*) = 0.06, *p* = 0.04; Table 8]. The four remaining tribal language activities (speaking tribal language to child, using tribal language in everyday life, using tribal language in prayers and songs, and speaking tribal language with other adults around the child) were not related to EF via either the Pencil Tap or the Leiter-R (Table 8).

**Table 8:** Specific language activities as predictors of the Leiter-R assessor-rated attention: hurdle models.

Model	Estimate	SE	95% CI		*p*
			LL	UL	
Exponential regression results					
Spoke Tribal lang. to child	0.11	0.06	−0.02	0.23	0.10
**Made sure child heard tribal lang.**	**0.14**	**0.06**	**0.01**	**0.27**	**0.04**
Encouraged child to Learn Tribal Lang.	−0.05	0.05	−0.17	0.07	0.37
Used Tribal lang. in Prayers/Songs with child	0.06	0.04	−0.03	0.14	0.18
Used Tribal lang. in Everyday Life with child	0.02	0.05	−0.08	0.13	0.64
Used Tribal lang. with other Adults around child.	0.10	0.05	−0.00	0.20	0.05
Logistic regression predicting hurdle (30)					
Spoke Tribal lang. to child	0.12	0.07	−0.04	0.27	0.13
**Made sure child heard tribal lang.**	**0.14**	**0.06**	**0.00**	**0.27**	**0.04**
Encouraged child to learn tribal lang.	0.11	0.06	−0.02	0.23	0.08
Used tribal lang. in prayers/songs with child	0.06	0.05	−0.05	0.17	0.27
Used tribal lang. in everyday life with child	0.07	0.08	−0.10	0.23	0.42
Used tribal lang. with other adults around child	0.08	0.07	−0.06	0.23	0.25

Separate regression analyses were ran for each cultural or language activity. All models account for sampling weights, clustering, and strata due to complex survey design, and controlled for age, gender, income, classroom cultural curriculum, classroom tribal language use, tribal home language, and neighborhood risk.

##### Follow-up analyses by tribal home language group.

At the request of AI/AN community, models investigating socialization through tribal language activities were analyzed separately for children whose caregivers who spoke their tribal language at home (43.8% of sample) versus those who did not. Results revealed that average use of tribal language activities only related to child attention on the Leiter-R attention for those whose parents/caregivers did *not* speak their tribal language at home. For these children, as average participation in language activities increased, children’s odds of belonging to the highest scoring group on the Leiter-R increased by 25.4% [95% CI (1.05, 1.40)]. In contrast, “encouraging children to learn their tribal language” remained a significant predictor of children’s EF scores on the Pencil Tap Task for all children, regardless of whether or not parent/caregiver tribal language use was included in the model. “Making sure children heard their tribal language” also significantly predicted EF scores on the Leiter-R for all children, but did so differently based on whether or not children’s parents/caregivers spoke a tribal language at home. For those whose parents did *not* speak their tribal language at home, “making sure children heard their tribal language” (by others) predicted passing the “hurdle” on the Leiter-R; these children had a 17.4% greater odds [95% CI (1.03, 1.34)] of belonging to the highest scoring group with every one level in frequency of hearing their tribal language (e.g., from “sometimes” to “often”). “Making sure their child heard their tribal language” also predicted higher attention ratings for children whose caregivers *did* speak their tribal language at home, given that children scored 0–29. For these children, as frequency of hearing their tribal language increased (e.g., from “sometimes” to “often”), their score on the Leiter-R increased by about one point 1.18 points (*SD* = 0.53, *p* = 0.01).

## Discussion

The current study investigated cultural socialization practices as sources of resilience for the development of EF for AI/AN children, and examined these associations in the context of perceived neighborhood risk. Results highlight several aspects of AI/AN cultural socialization as related to higher early EF skills. As expected, average participation in tribal language activities was associated with higher attention as measured by the Leiter-R (but was not related to the Pencil Tap). Participation in two specific language activities (making sure child heard their tribal language and encouraging child to learn their tribal language) and two cultural activities (playing AI/AN games and participating in ceremonies) also related to higher EF (see Results for details delineated by EF task). Contrary to hypotheses, sum cultural activities was not associated with EF as measured by either task. Despite higher adversity due to systemic and historic injustices for AI/AN communities (Markstrom et al., 2011; Burnette and Figley, 2016), families rated neighborhood risks as measured in the present study as relatively low, and contrary to hypotheses, neighborhood risk did not predict children’s EF as measured by either task. Although findings were not consistent across all measures, this study provides novel evidence that some aspects of AI/AN cultural socialization are associated with the development of early childhood EF, pointing to their potential role as connecting forces (essential resilience processes) that have the power to set the foundation for future healthy outcomes.

### Neighborhood risk and EF

Despite 42.5% of families meeting federal poverty level criteria, neighborhood risk showed no significant associations with EF. It is possible the measure lacked cultural relevance for AI/AN communities, as evidenced by the fact that respondents generally perceived most risks as either “not a problem” or only “somewhat of a problem.” Although measures in the current data were selected with input from Indigenous partners, they were likely limited by the dearth of AI/AN-specific measures of risk- emphasizing the need for research-community partnerships to explore the concept of “neighborhood risk” in tribal communities. For example, the following items are often understood to hang together to represent neighborhood risk in the general population: large distances from public services, poverty, lack of police, isolation, and lack of social connection. However, while tribal reservations are sometimes isolated and rural, with high poverty (Austin et al., 2020; Baldwin et al., 2020), community relationships are often strong and supportive. AI/AN children and families who reside in culturally homogenous communities also have better access to ceremonies, wisdom of elders, language revitalization, and ancestral lands. Indeed, families in the present study who reported higher neighborhood or community risk, and lower income, also reported engaging in *more* cultural and language socialization. These strong social and cultural connections may promote and protect health despite negative aspects of the neighborhood or community environment, but future research is needed to investigate this using measures that better reflect risk in the neighborhood or community environment for AI/AN tribal families.

The lack of significant associations between neighborhood risk and children’s EF also highlight a difference between the presence of adversity and subjective perceptions of risk. Evidence suggests ratings of *perceived* neighborhood risk are better predictors of health outcomes than objective ratings (Schaefer-McDaniel, 2009; French et al., 2014; Muñoz et al., 2020), likely because the impact of stressors on health depends on appraisal, especially when the stress exceeds coping resources and protective factors (Lazarus and Folkman, 1984; Sandi, 2013; Masten, 2018). This phenomenon is further illustrated by the fact that the present study (along with other recent studies in AI/AN communities; see Tsethlikai, 2011; Kim et al., 2022) did not find a relation between SES and childhood EF; a surprising finding considering studies with the general population have long affirmed a consistent link between lower SES and lower EF (Sektnan et al., 2010; Rea-Sandin et al., 2021). This conflict may exist because previous studies often examine between-group differences which cannot capture the variability in SES within one racial/ethnic community, as the present study was able to do.

### Cultural socialization and EF

#### Cultural activities

Sum cultural activities did not predict EF, possibly because the quality of cultural connections is more relevant for EF development than the quantity of activities in any given month. Two such specific activities that were significantly related to higher EF included playing AI/AN games and participating in tribal ceremonies. Both activities represent tangible ways children build strong and healthy connections to their culture, community, and spirituality, and both increase children’s connection to adults who can foster healthy EF through relationships with the children. AI/AN games differ by tribe, but often include games of chance and games of skill (Mississippi Valley Archaeology Center, n.d.). Examples of games of chance include dice games (traditionally made from bone, sticks, or stones), which weave together spiritual and cultural components with mathematical probability (Rauff, 2009). Games of skill focus on athleticism and include canoe races and stickball (lacrosse), a game which has many tribal origins. Prior to the “sportization” of the game through colonization, lacrosse had spiritual foundations, more freedom in the roles of players, less rigid time restrictions, and more emphasis on teamwork (Delsahut, 2015). Most AI/AN games, like many childhood games, require a set of general EF skills like close attention, cognitive flexibility to remember the rules, and inhibitory control over one’s own goals in favor of the team or partners. However, beyond additional cognitive skills, participating in tribal ceremonies (such as naming ceremonies and puberty ceremonies) plays a critical role in identity building for AI/AN children. Children disconnected from such ceremonies may not receive important building blocks on which to build their identity as members of their community. Additionally, participating in tribal ceremonies, as well as seasonal and land-based ceremonies, all serve to increase spiritual, intergenerational, and environmental connectedness. The present study aligns with Indigenous theoretical understandings of resilience, upholds cultural socialization as a potential “connecting force” (or resilience promoter; see Ullrich, 2019), and provides evidence these connecting forces may promote AI/AN children’s cognitive development.

#### Language activities

The present study also provides evidence that socialization through tribal language activities, a key component of Indigenous culture, may be associated with higher EF. Overall average language socialization predicted higher EF for AI/AN children, as did two specific language activities: encouraging children to learn their tribal language and making sure the children heard their tribal language. Tribal language learning may support cognitive outcomes like EF via multiple mechanisms. The neurological links between bilingualism and EF are controversial, but some evidence suggests that fluency in a second language is related to stronger EF (Bialystok and Barac, 2012; Rosselli et al., 2016; Choi et al., 2018). Regardless, learning one’s tribal language represents more than an additional cognitive skill. In AI/AN communities, knowing one’s tribal language may increase cultural efficacy, which in turn predicts connection, mental health, and thriving for Indigenous people (Gonzales et al., 2022). Knowing one’s tribal language also unites children with their culture, ceremonies, elders, and even ancestors. Thus, greater access to caring adults in the community may scaffold EF through social learning, a large contributor to EF in early childhood (Carlson, 2009).

The present study also followed community prompting to investigate the influence of language activities for families who did and did not speak their tribal language at home. First, many language socialization activities were significantly related to higher EF for parents/caregivers who *did* speak their tribal language, which speaks to the added value of socialization efforts beyond parents/caregivers’ own language use or abilities. Critically, results also showed that children received higher attention ratings when parents/caregivers did *not* speak their tribal language, but made efforts to expose their children to their tribal language (e.g., taking them to classes or having them listen to songs sung in their tribal language). It is also notable that many of the tribal language activities that demonstrated null findings with relation to children’s EF were activities which required parents/caregivers to speak their tribal language (e.g., “used tribal language in prayers and songs with child”). The implications of these findings are substantial, given over half of families in the present study (57.6%) did not speak their tribal language at home. This phenomenon reflects intergenerational loss stemming from impacts of the boarding school era (where tribal languages were banned; Campbell and Evans-Campbell, 2011) as well as ongoing experiences of racism that led to elders not being able to pass on their language to the next generation. More research is needed to fully investigate these patterns using more nuanced measures of tribal language fluency; levels of exposure within households, schools, and communities; and access to longitudinal data. Nevertheless, the present study yields promising evidence that revitalization efforts by tribes, parents, and communities- reclaiming culture and language for their children- may act as resilience factors and positively impact child cognitive health.

### Limitations and future directions

First, neighborhood risk was measured in the spring of preschool, several months after the cultural socialization measures. These surveys were spread across the preschool year to reduce participant burden and are used cohesively to represent a snapshot of the family environment during the preschool year, but concurrent measurement of these constructs should be considered in future studies. Second, the available data measured neighborhood risk using two scales with different response options, which led to the standardization and creation of the Neighborhood Risk Index in the present study. Although this adds important and novel information to the literature, future research should explore more nuanced approaches, such as factor analyses, to investigate patterns of neighborhood risk if using existing measures with AI/AN communities. However, while existing measures of risk represent a foundational starting point, items typically used to measure neighborhood risk in the general population may lack cultural relevance for AI/AN tribal communities. The construct of risk in the neighborhood or community environment for AI/AN communities remains poorly defined, and future research should explore other ways to measure this in tribal settings.

There were also some limitations with the EF measures. The Pencil Tap only assessed attention for children ages 4 years and older; thus 214 three-year old children (27.9%) were missing from this assessment. However, these children did not differ demographically from the full sample, and sensitivity analyses restricting the sample to those four and older yielded nearly identical results. Second, while the EF measures used in the present study were reliable and valid, it is possible that direct assessments performed by an outside researcher were unfamiliar for children in tribal communities. Most data collectors were non-Indigenous (Bernstein et al., 2018), and although they were well-trained, they may have interpreted cultural differences in children’s behavior (e.g., deferential respect for elders, observational learning style) as highly compliant or attentive. This may have contributed to the ceiling effect on the Leiter-R, although high scores on the Leiter-R are common in this age range (Raver et al., 2011; Mathematica Policy Research, 2012; Faldowski et al., 2013). Hurdle models, which we utilized, are designed to allow for analysis of such distributions (Hofstetter et al., 2016); and additional analyses with alternative statistical models (ZIP, censored regression) provided consistent results. Therefore, findings should be trustworthy given the current distribution, but because the Leiter-R did demonstrate such strong ceiling effects, it may not have captured the full range of underlying variability in attention. Results obtained using this measure are exploratory in nature and should be interpreted cautiously, but the Leiter-R provides important and novel information about how children’s attention skills are viewed (by the assessor) during structured tasks, complementing the Pencil Tap Task, which primarily assesses inhibitory control. Future researchers should partner with AI/AN communities to investigate cultural understandings of EF and explore culturally grounded ways to measure these skills observationally.

Last, the construct of “cultural socialization” is not yet well defined for AI/AN communities. In the present study, this was measured via participation in cultural activities (from a scale comprised of dichotomous (yes/no) variables), and tribal language activities. To contribute to the gap in the literature in this area, multiple models were run that tested the individual influence of specific cultural and language activities as related to children’s EF; however, it possible that the number of models run in the present study increased the probability of a Type I error. In addition, the phrasing of the questions, which asked parents/caregivers about socialization activities in the past month, may have led to under-reporting. For example, in the present study, nearly 20% of parents/caregivers reported engaging in zero of the six cultural activities with their children in the past month- yet activities like ceremonies, hunting, and gathering are often cyclical and participation may depend on season or tribe. Opportunities to examine tribe-specific cultural activities were limited by the need for a robust, nationally representative sample (which necessitated a “Pan-Indian” approach). While limiting, this approach aligns with guidelines such as the “Wheel of AI/AN specificity” (Walls et al., 2019) wherein research develops in an iterative fashion; moving from general AI/AN populations to tribe-specific, tailored measures. Finally, findings of the present study may not extend to all AI/AN children and families due to the sample population representing mostly reservation-based Head Start programs, which only serve 49% of all AI/AN youth that attend Head Start (Bernstein et al., 2018). Community-based, mixed-methods studies are needed to examine what cultural socialization and neighborhood risk looks like for urban AI/AN children and families.

### Implications

This study represents an important step forward toward culturally grounded evidence-based science to inform strategies to promote resilience with young AI/AN children. Overall, although findings were mixed, several cultural socialization practices by parents/caregivers emerged as related to higher EF for AI/AN preschool children. More specifically, findings regarding relations between AI/AN games, tribal language, and EF open the door for future research to design culturally based EF interventions that utilize AI/AN traditional games and language components. Existing interventions provide evidence that classroom games can be used to increase EF in preschool, including the Red Light, Purple Light intervention (Tominey and McClelland, 2011) and other team-based cognitively engaging physical activity interventions (Schmidt et al., 2015). Tribal Head Start programs are uniquely positioned to be able to implement such interventions as they already offer cultural curricula and resources to children and families. The present study also contributes to theory and measure development related to AI/AN cultural socialization. Previous studies with older children (approximately 9 to 12 years old) found that children defined their culture in terms of tangible, concrete aspects- including participating in traditional ceremonies, knowing their language, and eating traditional foods (Morris et al., 2002). Findings in the present study extend this literature to suggest that similarly tangible, concrete socialization activities like playing AI/AN games and learning tribal language may predict early cognitive skills like EF.

### Conclusion

Findings from the present study provide initial evidence that certain aspects of cultural socialization may promote resilience for AI/AN children through its relations with early EF skills. Although findings were not always consistent across the various measures, a clear pattern emerged that linked several cultural socialization practices with higher EF skills for AI/AN preschoolers. The strongest evidence was detected for tribal language use: average participation in language activities, as well as two specific language activities (making sure the child heard their tribal language and encouraging the child to learn their tribal language) predicted at least one of the two EF measures. Additionally, two cultural activities (playing AI/AN games and participating in ceremonies), but not cumulative cultural activities, were associated with higher EF. In contrast, results indicated that neighborhood risk showed no relation with EF, which in addition to lack of variability, may indicate that this measure fails to capture adversity for this population. The present study affirms emerging Indigenous theories related to resilience (e.g., Ullrich, 2019), which emphasize the power of “connecting forces” in promoting health for AI/AN children. Learning tribal languages, participating in ceremonies, and playing traditional games all connect children to those that came before them, re-forging a community connection that has been in continuum since time immemorial, despite disconnecting forces that have caused adversity for AI/AN communities (e.g., poverty, historical trauma). The present study lays groundwork for future partnerships between tribes, researchers, policymakers, and practitioners to design interventions that increase cultural connectedness and EF in order to foster a wide range of healthy outcomes for generations to come.

## Data availability statement

The data analyzed in this study is subject to the following licenses/restrictions: data cannot be accessed without application and approval from the American Indian/Alaska Native FACES data committee. Requests to access these datasets should be directed to https://www.childandfamilydataarchive.org/cfda/archives/cfda/studies/36804.

## Ethics statement

The studies involving humans were approved by the Oregon State University Human Research Protection Program and Institutional Review Board. The study was determined to meet the definition of research but did not involve human subjects under the regulations set forth by the Department of Health and Human Services 45 CFR 46. The studies were conducted in accordance with the local legislation and institutional requirements. Written informed consent for participation was not required from the participants or the participants’ legal guardians/next of kin in accordance with the national legislation and institutional requirements.

## Author contributions

AM: Conceptualization, Funding Acquisition, Investigation, Methodology, Formal Analysis, Writing – original draft, Writing- review & editing. SL: Conceptualization, Supervision, Writing – review & editing. MM: Conceptualization, Supervision, Writing – review & editing. GG: Conceptualization, Supervision, Formal analysis, Methodology, Writing – review & editing. MT: Conceptualization, Methodology, Resources, Supervision, Writing – review & editing.
