# Corrigendum: Nurturing resilience in American Indian/Alaska Native preschool children: the role of cultural socialization, executive function, and neighborhood risk

**DOI:** 10.3389/fpsyg.2024.1364690

**Published:** 2024-01-11

**Authors:** Alexis Merculief, Shannon Lipscomb, Megan M. McClelland, G. John Geldhof, Monica Tsethlikai

**Affiliations:** ^1^Human Development and Family Studies, Oregon State University, Corvallis, OR, United States; ^2^Sanford School of Social and Family Dynamics, Arizona State University, Tempe, AZ, United States

**Keywords:** cultural socialization, language socialization, resilience, executive function, preschool, neighborhood risk, Indigenous, AI/AN

In the published article, there was an error in affiliation 1. Instead of “Human Development and Family Studies, Stanford University, Stanford, CA, United States” it should be “Human Development and Family Studies, Oregon State University, Corvallis, OR, United States”.

The authors apologize for this error and state that this does not change the scientific conclusions of the article in any way. The original article has been updated.

